# AmpSeqR: an R package for amplicon deep sequencing data analysis

**DOI:** 10.12688/f1000research.129581.2

**Published:** 2025-10-17

**Authors:** Jiru Han, Jacob E. Munro, Melanie Bahlo

**Affiliations:** 1 Population Health and Immunity Division, The Walter and Eliza Hall Institute of Medical Research, Melbourne, VIC, 3052, Australia; 2 Department of Medical Biology, The University of Melbourne, Melbourne, VIC, 3052, Australia

**Keywords:** amplicon sequencing, data visualization, summary report, R package

## Abstract

Amplicon sequencing (AmpSeq) is a methodology that targets specific genomic regions of interest for polymerase chain reaction (PCR) amplification so that they can be sequenced to a high depth of coverage. Amplicons are typically chosen to be highly polymorphic, usually with several highly informative, high frequency single nucleotide polymorphisms (SNPs) segregating in an amplicon of 100–200 base pair (bp). This allows high sensitivity detection and quantification of the frequency of each sequence within each sample making it suitable for applications such as low frequency somatic mosaicism detection or minor clone detection in mixed samples. AmpSeq is being increasingly applied to both biological and medical studies, in applications such as cancer, infectious diseases and brain mosaicism studies. Current bioinformatics pipelines for AmpSeq data processing lack downstream analysis, have difficulty distinguishing between true sequences and PCR sequencing errors and artifacts, and often require bioinformatic expertise. We present a new R package: AmpSeqR, designed for the processing of deep short-read amplicon sequencing data, with a focus on infectious diseases. The pipeline integrates several existing R packages combining them with newly developed functions to perform optimal filtering of reads to remove noise and improve the accuracy of the detected sequences data, permitting detection of very low frequency clones in mixed samples. The package provides useful functions including data pre-processing, amplicon sequence variants (ASVs) estimation, data post-processing, data visualization, and automatically generates a comprehensive Rmarkdown report that contains all essential results facilitating easy inclusion into reports and publications. AmpSeqR is publicly available at https://github.com/bahlolab/AmpSeqR.

## Introduction

Amplicon sequencing (AmpSeq) is increasingly used in biological and medical studies, offering high coverage for the detection of rare variants and multiplexing capacity that reduces sequencing costs. It is highly scalable and cost-effective, enabling data generation for large sample sizes, and can be further multiplexed at the marker level to increase resolution. Severe acute respiratory syndrome coronavirus 2 (SARS-CoV-2), the causative agent of coronavirus disease 2019 (COVID-19), continues to pose a serious threat to the global population health. While whole-genome sequencing was commonly used for SARS-CoV-2, targeted AmpSeq of defined genome regions was also widely applied, particularly in diagnostic and surveillance settings, to monitor viral presence and detect emerging variants. ^1^^,^^2^ AmpSeq methods are widely used in characterization and surveillance studies of many other infectious diseases (e.g., malaria infection) to monitor the emergence and spread of drug resistance, ^3^^–^^7^ to examine population structure, ^8^^,^^9^ to study relapse in malaria caused by activation of dormant liver-stage hypnozoites, ^10^ to estimate clearance rates ^11^ and to track within-host dynamics in longitudinal studies. ^12^

There are several bioinformatics pipelines for processing AmpSeq data, such as DADA2, ^13^ SeekDeep, ^14^ and HaplotypR. ^15^ SeekDeep and HaplotypR were specifically developed and tailored to study *Plasmodium* spp. However, these pipelines have some limitations, such as a lack of downstream analysis, difficulty in distinguishing between true sequence and PCR sequencing errors and artifacts such as chimeric reads or spurious low-frequency variants, and often requiring extensive bioinformatics expertise. We introduce AmpSeqR, a freely available R package ( https://github.com/bahlolab/AmpSeqR) that performs AmpSeq data analysis. The pipeline integrates several R packages as well as newly developed functions to filter out sequencing noise, including the removal of chimeric reads, correction of indels and homopolymers, checks of sequence similarity against the reference, evaluation of variant heterozygosity, and dataset-level checks, all of which improve the accuracy of sequence data detection. The pipeline offers various analysis steps including data pre-processing, amplicon sequence variants (ASVs) estimation, data post-processing, data visualization, and automatically generates a comprehensive report in Rmarkdown that contains all essential results. This pipeline is designed to simplify bioinformatics processing leading to a comprehensive pipeline that starts from raw FASTQ files and finishes by generating a final reproducible report, from a reproducible pipeline. We apply AmpSeqR to various AmpSeq datasets, including the SARS-CoV-2 dataset and malaria parasite *Plasmodium falciparum* datasets.

## Methods

### Implementation

The AmpSeqR package was built in R and is hosted on GitHub. It can be installed from https://github.com/bahlolab/AmpSeqR. The input files for AmpSeqR are the standard paired-end FASTQ format provided by the common Illumina sequencing platforms (e.g., MiSeq), as well as sample barcodes and target amplicon details.

### Operation

**Figure 1.**
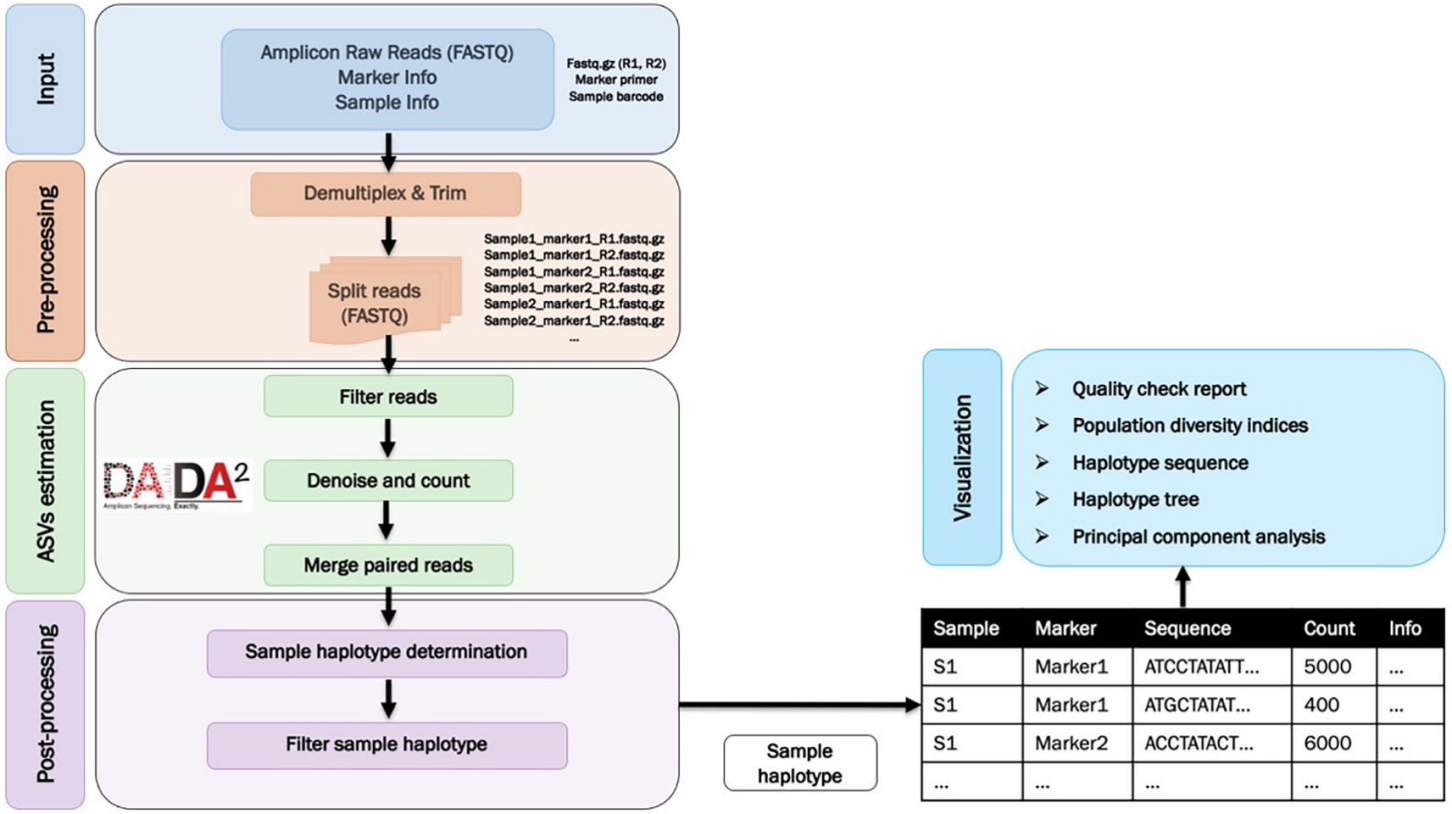
A flow chart summarising the AmpSeqR workflow which consists of four main steps.

AmpSeqR was developed and tested on R (version 4.1.3) with several other dependencies and the base functions in R and is compatible with Mac OS X, Windows, and major Linux operating systems. AmpSeqR is maintained at GitHub ( https://github.com/bahlolab/AmpSeqR). The archived source code can be found in https://doi.org/10.5281/zenodo.7580184. ^16^ AmpSeqR uses Biostrings ^17^ (v2.62.0, RRID:SCR_016949), ShortRead ^18^ (v1.52.0, RRID:SCR_006813), DADA2 ^13^ (v1.20.0), DECIPHER ^19^ (v2.20.0, RRID:SCR_006552), rlang (v1.0.2), Rmarkdown (v2.13), gtools (v3.9.2), withr (v2.5.0), utils (v4.1.1), cowplot (v1.1.1), furrr (v0.2.3), future (v1.24.0), GenomicRanges ^20^ (v1.46.1), VariantAnnotation ^21^ (v1.38.0), S4Vectors (v0.32.3), IRanges ^20^ (v2.28.0), plyr (v1.8.7), DT (v0.22), plotly (v4.10.0, RRID:SCR_013991), viridisLite (v0.4.0), flextable (v0.7.0), scales (v1.2.0, RRID:SCR_019295), magrittr (v2.0.3), heatmaply (v1.3.0), digest (v0.6.29), tidyselect (v1.1.2), data.table (v1.14.2), ComplexHeatmap ^22^ (v2.11.1, RRID:SCR_017270), htmltools (v0.5.2), ape (v5.6-2, RRID:SCR_017343), randomcoloR (v1.1.0.1), ggtree ^23^ (v3.0.4, RRID:SCR_018560), ggstance (v0.3.5), tibble (v3.1.8), stats (v4.1.3) and several tidyverse packages (v1.3.1, RRID:SCR_019186). The workflow is illustrated in Figure 1.

*Data pre-processing*

The data pre-processing step demultiplexes the raw paired-end FASTQ reads and trims the sample barcodes and target amplicon primer sequences as well as assigns the sequence to each sample and marker (amplicon target) combination. As each individual has a unique oligonucleotide barcode and each marker has a unique primer sequence distinguishing it from other markers, sequence reads can be de-multiplexed to separate individual samples and different markers, followed by trimming the primer sequence. The data pre-processing is handled using the Biostrings, ShortRead, rlang, magrittr, future, and tidyverse R packages.

*Amplicon sequence variants (ASVs) estimation*

The amplicon sequence variants (ASVs) estimation process identifies amplicon haplotype sequences. We leveraged several functions in the DADA2 R package, such as *filterAndTrim*, *derepFastq*, *learnErrors*, *dada*, and *mergePairs* to obtain the haplotype sequence. DADA2 can handle sequences with indels and accurately determines sequence variants leading us to choose DADA2 as the preprocessing tool to call ASVs in our AmpSeqR pipeline. For merging of paired-end reads we provided an option that can process the paired-end reads without any overlap. We also added some functions based on ShortRead, future, and tidyverse R packages for automatic downsampling of each sample/amplicon combination to a certain number of reads (e.g., 10,000) to reduce the computation time required. This step reduces excessive coverage and computational cost. The GATK (RRID:SCR_001876) documentation states that having additional data is not informative when read coverage exceeds a certain depth. A coverage of 10,000 reads was found to be sufficient to detect minor clones with a frequency as low as 0.1%. ^15^

*Data post-processing*

This step primarily optimises amplicon haplotype calling through chimeric read detection and removal and variant filtration. The removal of sequencing background noise such as ultra-rare variants and chimeric reads generated during PCR amplification are important steps for the generation of high fidelity amplicon sequencing data. Chimeric reads are sequences formed from two or more biologically distinct sequences joined together during PCR amplification. Chimeric reads are much more frequent in amplicon sequencing applications in comparison to whole-exome sequencing (WES) or whole genome sequencing (WGS) because the same as well as very similar sequences appear in increased abundance. ^24^ In addition, there are several types of indel errors in short-read Illumina sequencing data, such as homopolymers and terminal indels, which are the main source of low-quality indel calls. ^25^ Several parameters are used to filter haplotypes, such as: (i) haplotype frequency, (ii) sequence similarity (haplotype sequences were mapped against reference sequence for each amplicon marker to calculate the sequence similarity), as well as (iii) variant heterozygosity, and (iv) minor allele frequency (MAF) in a given dataset. The detailed parameter options and their default values are listed in Table 1. After removing the background noise, the final haplotypes for each sample and marker combination are identified. This step is handled using the Biostrings, DECIPHER, rlang, withr, GenomicRanges ^20^ (RRID:SCR_000025), VariantAnnotation ^21^ (RRID:SCR_000074), S4Vectors, IRanges ^20^ (RRID:SCR_006420), Parallel, gtools, utils, magrittr, tibble, and tidyverse R packages.

**Table 1.** Parameters used to filter haplotypes in AmpSeqR.

Option	Default	Description
min_read_count	1000	The minimum number of reads per sample per marker.
min_marker_count	100	The minimum number of reads for each amplicon marker after calling haplotype.
n_sample	10000	Downsamples an exact number of reads from paired-end FASTQ files.
min_overlap	10	The minimum overlap length for merging paired-end reads.
min_asv_count	5	The minimum number of reads for each haplotype.
min_asv_freq	0.001	The minimum haplotype frequency.
min_ident	0.75	The minimum sequence similarity.
min_ident_z	-3	The minimum standardized sequence similarity.
max_breakpoints	3	The maximum number of breakpoints in chimeric reads.
min_parent_ratio	1.5	The minimum parent:child ratio for chimeric sequence detection.
max_sm_miss	0.5	The maximum fraction of missing data for samples.
max_marker_miss	0.5	The maximum fraction of missing data for amplicon markers.
min_homo_rep	3	The minimum length of the homopolymer repeats
sample_med_He	0	The maximum median expected heterozygosity (a measure of genetic variation within populations) for the variance of all samples in the dataset.
n_alleles	3	The maximum number of alleles at a locus.
var_maf	0.001	The minimum minor allele frequency of the variant.
var_he	0.001	The minimum expected heterozygosity of the variant.

*Data visualization*

The data visualization step generates a comprehensive Rmarkdown report that contains various summary data visualizations and data summaries, such as quality checks, performed at both the individual/sample and amplicon level, amplicon haplotype sequence counts, haplotype diversity metrics of amplicon markers in the dataset, haplotype sequence visualization, haplotype tree generation, and principal component analysis (PCA). Quality checks include checking sample and amplicon marker information, an overview of read counts to track reads at various points in the pipeline, missing values in the dataset, and passed samples and markers. Haplotype diversity metrics such as expected heterozygosity (He), number of single nucleotide polymorphisms (SNPs), and number of unique haplotypes detected per sample per marker, are generated to help explore the amplicon marker diversity in the dataset. Detected haplotype sequences can be aligned to the reference genome and visualized to explore SNP positions and nucleotide changes. The haplotype tree is created by integrating all major haplotype sequences (>50% relative abundance) in each sample and performing multiple sequence alignment to identify the similarity between samples. PCA is also performed to visualize the sample structure. More broadly, the data visualization and reporting steps in AmpSeqR are implemented using a range of R packages, including Biostrings, Rmarkdown, plyr, DT, plotly, viridisLite, stats, flextable, ComplexHeatmap, ^22^ scales, heatmaply, digest, tidyselect, data.table, DECIPHER, htmltools, pheatmap (RRID:SCR_016418), ape, randomcoloR, ggtree, ^23^ ggstance, and several tidyverse R packages.

The major functions of the AmpSeqR package are summarised in Table 2.

**Table 2.** Description of the AmpSeqR major functions.

Function	Description	Key R packages implemented
demultiplex_reads	Demultiplexing and truncating sample barcodes and primer sequence	Biostrings, ShortRead, magrittr, parallel, purrr
plot_quality	Visualize the quality profiles of the forward and reverse reads of amplicon markers	ShortRead, tidyverse, cowplot
dada_filter	Filters poor quality reads from the input FASTQ files	dada2, tidyverse, magrittr, purrr
downsample_reads	Downsampling per sample per amplicon data to a certain number of reads	ShortRead, future
dada_seq_tbl	Merge paired-end reads and construct an ASV table	dada2, DECIPHER, Biostrings, parallel, future, tidyverse
annotate_seq_tbl	Sample haplotype sequence determination (True or noise)	DECIPHER, Biostrings, future, furrr, tidyverse
clean_homopolymers	Detect indels in homopolymers and change the indel to be the same as in the reference genome	DECIPHER, Biostrings, tidyverse
clean_terminal_indels	Change terminal indels to be the same as in the reference genome	DECIPHER, Biostrings, tidyverse
sequence_filter	Filter haplotype sequences	tidyverse, purr, GenomicRanges, VariantAnnotation, S4Vectors, IRanges
generate_report	Generate a comprehensive Rmarkdown report	rmarkdown, DT, plotly, ComplexHeatmap, heatmaply, DECIPHER, Biostrings, ape, ggtree, htmltools, pheatmap, randomcoloR, randomcoloR, tidyverse
process_run	Runs all functions to quickly generate the results	all of the above packages

We also provide benchmarking results to illustrate the runtime performance of these functions across different datasets ( Table 3).

**Table 3.** Runtime performance of AmpSeqR functions across datasets of different size and complexity. All values are reported in seconds.

Function	Dataset 1 Synthetic *P. falciparum* AmpSeq (5 samples, 2 markers, 100k reads)	Dataset 2 SARS-CoV-2 AmpSeq (Demultiplexed) (19 samples, 8 markers, 1.0M reads)	Dataset 3 *P. falciparum* AmpSeq (16 samples, 2 markers, 415k reads)	Dataset 4 In-house Large Dataset (231 samples, 12 markers, 22.6M reads)
demultiplex_reads	25.612	-	48.743	5182.284
dada_filter	3.383	16.3	10.697	978.675
downsample_reads	0.002	8.88	7.767	89.723
dada_seq_tbl	0.762	10.427	8.945	1179.455
annotate_seq_tbl	2.052	0.908	7.378	121.851
sequence_filter	2.991	3.368	0.981	80.157
generate_report	16.816	15.897	6.095	120.125
process_run	33.602	-	80.828	1.126e+04

## Use cases

In this section, we demonstrate the application of AmpSeqR for the data referenced in Datasets.

### Datasets

Three datasets are used for the Use cases section. Dataset 1 is provided with the package and datasets 2 and 3 need to be downloaded via the published papers in which they appeared.

*Dataset 1: Synthetic Plasmodium falciparum AmpSeq data*

The synthetic *P. falciparum* paired-end amplicon sequencing data (FASTQ) was generated using the ART ^26^ and ShortRead ^18^ packages. The dataset consists of five *P. falciparum* samples with defined mixtures (1:1, 1:10, 1:100, 1:500, 1:1000) of two *P. falciparum* reference genomes *Pf3D*7 and *PfDd2* (downloaded from PlasmoDB). We selected two amplicon markers, the *P. falciparum* surface marker genes circumsporozoite protein (CSP) and thrombospondin-related anonymous protein (TRAP). The FASTQ sequence data, as well as additional amplicon primer and sample barcode details are included in the AmpSeqR package.

*Dataset 2: SARS-CoV-2 AmpSeq data*

The dataset for multiplexed SARS-CoV-2 was obtained through the Aynaud *et al.* ^1^ study. We downloaded the PoC cohort, which includes 19 nasopharyngeal swab samples, of which 17 were positive for SARS-CoV-2. This study targeted six amplicon markers: one human gene Peptidylprolyl Isomerase B (PPIB) as a quality control, and five SARS-CoV-2 regions targeting the Nucleocapsid (N), Envelope (E), RNA-dependent RNA polymerase (RdRP), and two regions of the Spike (S) gene that correspond to the receptor-binding domain (RBD) and the polybasic cleavage site (PBS). Amplicon primer details are given in Aynaud *et al.* ^1^ Sequencing was performed using the Illumina MiSeq sequencing platform. This dataset and metadata are available in Gene Expression Omnibus (GEO) at NCBI (GEO accession number GSE160031). The complete genome SARS-CoV-2 isolate Wuhan-Hu-1 (NC_045512) was used as the reference genome for sequence analysis and was downloaded from NCBI ( NC_045512.2).

*Dataset 3: Plasmodium falciparum AmpSeq data*

The *P. falciparum* dataset can be downloaded from the Lerch *et al.* ^15^ study. The dataset consists of 16 defined mixtures of *P. falciparum* genomic DNA samples, where the mixtures of two *P. falciparum* lab strains HB3 and 3D7 were combined in various proportions (1:1, 1:10, 1:50, 1:100, 1:500, 1:1000, 1:1500, and 1:3000). Lerch *et al.* targeted two *P. falciparum* surface marker genes: (i) the conserved *Plasmodium* membrane protein (CPMP) and, (ii) the circumsporozite protein (CSP). This dataset is available on the NCBI-SRA website: BioProject accession PRJNA381546. Amplicon primer and sample barcodes details are given in the Lerch *et al.* study and can be downloaded from https://github.com/lerch-a/HaplotypR.git. This provides a test dataset to evaluate the detectability of minority clones of AmpseqR.

### Installing the package

The AmpSeqR package is hosted in GitHub and can be installed with the following command line.

devtools::install_github("bahlolab/AmpSeqR")



Load the AmpSeqR package into the workspace as well as other packages required for the following analysis.

library(AmpSeqR)
library(tidyverse)



Here we present the AmpSeqR workflow with a minimal example dataset (example_data). The input files are the standard paired-end FASTQ files provided by the Illumina sequencing platforms, as well as sample barcodes and target amplicon details. The sample barcodes file should include sample_id, barcode_fwd, barcode_rev, sample (sample name, can be the same as sample_id), info (e.g., sample type). All columns should be in character format, and barcode_fwd and barcode_rev must be consistent DNA-encoded barcodes of equal length. The target amplicon detail file should include marker_id, primer_fwd, primer_rev, seq (reference sequence), chrom (chromosome), start (reference sequence start position), end (reference sequence end position). All columns should be in character format, except start and end, which must be numeric. This file should be set up in a text editor or excel and saved as text CSV format (*.csv) file.

example_data <- get_ampseqr_example_data()
example_data

## $marker_info
## # A tibble: 2 × 7
##  marker_id primer_fwd      primer_rev seq   chrom  start   end
##  <chr>   <chr>         <chr>    <chr>  <chr>  <int>   <dbl>
## 1  CSP    GTGGAGTATGTCCGTAACT… CTAGCA…   CCCA…  Pf3D…  222253  222372
## 2  TRAP   CTTCTACGTCTTACAAAGG… CGTGAT…   TGGG…  Pf3D…  1465085 1465204
##
## $sample_manifest
## # A tibble: 5 × 5
##  sample_id barcode_fwd barcode_rev sample         info
##  <chr>   <chr>     <chr>     <chr>          <chr>
## 1 S01     ATCGCTAT   TCTAATGT   3D7_Dd2_1_1       1_1
## 2 S02     GTGACGAA   TTACAGCA   3D7_Dd2_1_10       1_10
## 3 S03     CTATTGCA   GTGCTAAT   3D7_Dd2_1_100     1_100
## 4 S04     CGATCGAT   GCACTTGT   3D7_Dd2_1_500      1_500
## 5 S05     AGAGGGAC   GCAAGCGT   3D7_Dd2_1_1000     1_1000
##
## $reads_2
## [1] "~/R/x86_64-pc-linux-gnu-library/4.1/AmpSeqR/extdata/readsR.fastq.gz"
##
## $reads_1
## [1] "~/R/x86_64-pc-linux-gnu-library/4.1/AmpSeqR/extdata/readsF.fastq.gz"
##
## $data_dir
## [1] "~/R/x86_64-pc-linux-gnu-library/4.1/AmpSeqR/extdata"



### Data pre-processing

This step demultiplexes the raw paired-end FASTQ reads and trims the sample barcodes and target amplicon primer sequences and assigns reads to each sample and each amplicon marker combination.

Here, we first use the synthetic *Plasmodium falciparum* AmpSeq data.

*# Input data*
reads_1 <- example_data$reads_1
reads_2 <- example_data$reads_2
sample_manifest <- example_data$sample_manifest
marker_info <- example_data$marker_info



The analysis directory is then created.

*# Create the analysis directory*
dir.create("./runs")
run_dir <- "runs"



Next, we demultiplex the reads using the demultiplex_reads function as follows.

*# Demultiplex reads*
demultiplexed <- demultiplex_reads(sample_manifest = sample_manifest,
                     marker_info = marker_info,
                     reads_1 = reads_1,
                     reads_2 = reads_2,
                     output_dir = run_dir,
                     output_sub_dir = file.path (run_dir, 'demultiplex'))

## note: 100000 of 100000 reads demultiplexed successfully.

demultiplexed
## # A tibble: 10 × 7
##  sample_id marker_id reads_1   reads_2  n     sample     info
##  <chr>   <chr>   <chr>    <chr>    <int>   <chr>      <chr>
## 1 S01     CSP    /storn…   /storn…   10000   3D7_Dd2…    1_1
## 2 S01     TRAP    /storn…   /storn…   10000   3D7_Dd2…    1_1
## 3 S02     CSP    /storn…   /storn…   10000   3D7_Dd2…    1_10
## 4 S02     TRAP    /storn…   /storn…   10000   3D7_Dd2…    1_10
## 5 S03     CSP    /storn…   /storn…   10000   3D7_Dd2…    1_100
## 6 S03     TRAP    /storn…   /storn…   10000   3D7_Dd2…    1_100
## 7 S04     CSP    /storn…   /storn…  10000   3D7_Dd2…    1_500
## 8 S04     TRAP    /storn…   /storn…  10000   3D7_Dd2…    1_500
## 9 S05     CSP    /storn…   /storn…   10000   3D7_Dd2…    1_1000
## 10 S05     TRAP    /storn…   /storn…  10000   3D7_Dd2…    1_1000



The main outputs from the demultiplex_reads function are:

• demultiplex folder: demultiplexed paired-end FASTQ files.
• demultiplex.rds: the demultiplexed table in RDS format which includes sample_id, marker_id, reads_1 (the forward read FASTQ file path), reads_2 (the reverse read FASTQ file path), n (number of demultiplexed reads), sample, info.

**Figure 2.**
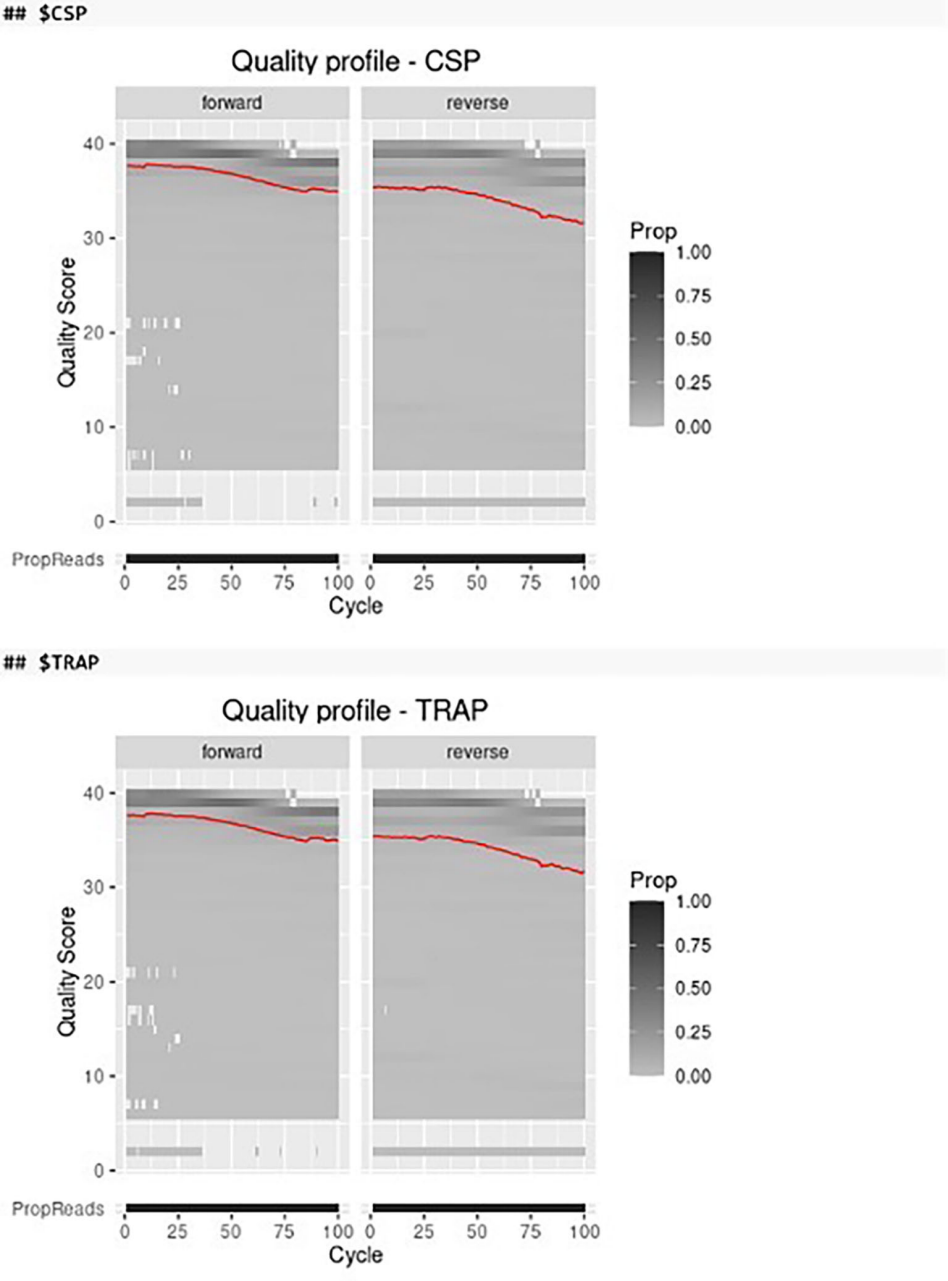
The quality profile of the forward and reverse reads. The red line shows the mean quality score of all samples in each cycle. Color represents the proportion of reads of each quality score in each cycle.

Before filtering and trimming the paired-end FASTQ files we can visualize the quality profiles of the forward and reverse reads of different amplicon markers by using the plot_quality function in AmpSeqR which integrates several functions from the ShortRead, DADA2, cowplot and tidyverse packages ( Figure 2).

*# Visualize the quality profiles of the forward and reverse reads*
read_quality <- plot_quality(demultiplexed)
read_quality

## $CSP


## $TRAP



According to the quality profiles of the forward and reverse reads, we can define the trim position for the forward and reverse reads to remove low-quality bases. The marker_trim should be a data frame with three columns marker_id (character), trim_fwd (integer), and trim_rev (integer), or can be left unset (NULL, run without trimming).

The next step filters the poor-quality reads and trim low-quality bases as follows. Filtering is implemented using the dada_filter function, which uses the filterAndTrim function from the DADA2 package.

*# Filter and trim reads*
flt_reads <- demultiplexed %>%
dada_filter(output_dir = run_dir,
      output_sub_dir = file.path(run_dir, 'filter'))
flt_reads

## # A tibble: 10 × 9
##   sample_id marker_id n   sample  info  reads_1  reads_2   n_in   n_out
##   <chr>    <chr>   <int> <chr>   <chr>  <chr>    <chr>   <dbl>  <dbl>
## 1 S01     CSP     10000 3D7_Dd2… 1_1   /storn…   /storn…  10000 4878
## 2 S01     TRAP    10000 3D7_Dd2… 1_1   /storn…   /storn…  10000 4876
## 3 S02     CSP     10000 3D7_Dd2… 1_10  /storn…   /storn…  10000 4874
## 4 S02     TRAP    10000 3D7_Dd2… 1_10  /storn…   /storn…  10000 4869
## 5 S03     CSP     10000 3D7_Dd2… 1_100   /storn…   /storn…  10000 4818
## 6 S03     TRAP    10000 3D7_Dd2… 1_100   /storn…   /storn…  10000 4868
## 7 S04     CSP     10000 3D7_Dd2… 1_500   /storn…   /storn…  10000 4935
## 8 S04     TRAP    10000 3D7_Dd2… 1_500   /storn…   /storn…  10000 4933
## 9 S05     CSP     10000 3D7_Dd2… 1_1000  /storn…   /storn…  10000 4740
## 10 S05     TRAP    10000 3D7_Dd2… 1_1000  /storn…   /storn…  10000 4953



The main outputs for the dada_filter function are:

• a filter folder: filtered and trimmed paired-end FASTQ files.
• a filtered_reads.rds table: the filtered table in RDS format which includes sample_id, marker_id, n (number of demultiplexed reads), sample, info, reads_1 (the filtered and trimmed forward FASTQ file path), reads_2 (the filtered and trimmed reverse FASTQ file path), n_in (number of reads before filtering and trimming), n_out (number of reads after filtering and trimming).

The downsample_reads function randomly downsamples each sample dataset to a pre-specified number of reads (default, 10,000 reads) to reduce the computation time.

*# Downsampling reads*
sub_reads <- flt_reads %>%
  downsample_reads(output_dir = run_dir,
            output_sub_dir = file.path(run_dir, 'downsample'),
            count_col = 'n_out')
sub_reads

## # A tibble: 10 × 8
##   sample_id marker_id sample   info   reads_1  reads_2   n_out  n
##   <chr>   <chr>    <chr>   <chr>   <chr>    <chr>     <dbl>  <dbl>
## 1 S01    CSP     3D7_Dd2…  1_1     /storn…  /storn…   4878   4878
## 2 S01     TRAP    3D7_Dd2…  1_1    /storn…  /storn…   4876   4876
## 3 S02    CSP     3D7_Dd2…  1_10   /storn…  /storn…   4874   4874
## 4 S02     TRAP    3D7_Dd2…  1_10   /storn…  /storn…   4869   4869
## 5 S03    CSP     3D7_Dd2…  1_100  /storn…  /storn…   4818   4818
## 6 S03     TRAP    3D7_Dd2…  1_100  /storn…  /storn…   4868   4868
## 7 S04    CSP     3D7_Dd2…  1_500  /storn…  /storn…   4935   4935
## 8 S04     TRAP    3D7_Dd2…  1_500  /storn…  /storn…   4933   4933
## 9 S05    CSP     3D7_Dd2…  1_1000  /storn…  /storn…   4740   4740
## 10 S05     TRAP    3D7_Dd2…  1_1000  /storn…  /storn…   4953   4953



The main outputs for the downsample_reads function are:

• the downsample folder: downsampled paired-end FASTQ files with a read number greater than n_sample.
• the subsampled_reads.rds table: the downsampled table in RDS format includes sample_id, marker_id, sample, info, reads_1 (the downsampled forward FASTQ file path), reads_2 (the downsampled reverse FASTQ file path), n_out (number of reads before downsampling), n (number of reads after downsampling).

### Amplicon sequence variants (ASVs) estimation

The dada_seq_tbl function use several functions in DADA2, Biostrings, DECIPHER, and Parallel packages, such as *derepFastq*, *learnErrors*, *dada*, *mergePairs*, *AlignSeqs*, *DistanceMatrix* to filter noise reads and merge the paired-end reads and obtain the amplicon haplotype sequence. When paired-end reads do not overlap, the min_overlap parameter should be set to -1.

*# Construct amplicon sequence variant table (ASV) table*
seq_tbl <- sub_reads %>%
  dada_seq_tbl(output_dir = run_dir)
seq_tbl

## # A tibble: 19 × 7
##   sample_id  marker_id sequence         count  status sample info
##   <chr>    <chr>    <chr>           <int>   <chr>  <chr>   <chr>
## 1 S01     CSP      CCCAAATGCAAACCCAAATG… 2481   pass  3D7_D…  1_1
## 2 S01     CSP      CCCCAATGCAAACCCAAATG… 2397   pass  3D7_D…  1_1
## 3 S01     TRAP     TGGGTGAACCATGCAGTACC… 2444   pass  3D7_D…  1_1
## 4 S01     TRAP     TGGGTGAAGCATGCAGTACC… 2432   pass  3D7_D…  1_1
## 5 S02     CSP      CCCAAATGCAAACCCAAATG…  496   pass   3D7_D…  1_10
## 6 S02     CSP      CCCCAATGCAAACCCAAATG… 4378   pass  3D7_D…  1_10
## 7 S02     TRAP     TGGGTGAACCATGCAGTACC…  495   pass  3D7_D…  1_10
## 8 S02     TRAP     TGGGTGAAGCATGCAGTACC… 4374   pass  3D7_D…  1_10
## 9 S03     CSP      CCCAAATGCAAACCCAAATG…    40   pass   3D7_D…  1_100
## 10 S03     CSP      CCCCAATGCAAACCCAAATG… 4778   pass  3D7_D…  1_100
## 11 S03      TRAP     TGGGTGAACCATGCAGTACC…    63   pass   3D7_D…  1_100
## 12 S03     TRAP     TGGGTGAAGCATGCAGTACC… 4805   pass  3D7_D…  1_100
## 13 S04     CSP      CCCAAATGCAAACCCAAATG…   9   pass  3D7_D…  1_500
## 14 S04     CSP      CCCCAATGCAAACCCAAATG… 4926   pass  3D7_D…  1_500
## 15 S04     TRAP     TGGGTGAACCATGCAGTACC…   8   pass  3D7_D…  1_500
## 16 S04     TRAP     TGGGTGAAGCATGCAGTACC… 4925   pass  3D7_D…  1_500
## 17 S05     CSP      CCCCAATGCAAACCCAAATG… 4737  pass  3D7_D…  1_10…
## 18 S05     TRAP     TGGGTGAACCATGCAGTACC…   5   pass  3D7_D…  1_10…
## 19 S05     TRAP     TGGGTGAAGCATGCAGTACC… 4948  pass  3D7_D…  1_10…



The main outputs for the dada_seq_tbl function are:

• the seq_tbl.rds table: the amplicon haplotype sequence table in RDS format includes sample_id, marker_id, sequence (haplotype sequence), count (read counts), status (initial haplotype status), sample, info.

### Data post-processing

The annotate_seq_tbl function leverages various useful functions in Biostrings and DECIPHER to determine the amplicon haplotype status and to distinguish the true haplotype sequences from sequencing background noise and artifacts, including ultra-rare variants and chimeric reads generated during PCR amplification.

*# Haplotype inference*
seq_ann_tbl <- seq_tbl %>%
annotate_seq_tbl(marker_info = marker_info,
         output_dir = run_dir)
seq_ann_tbl

## # A tibble: 19 × 9
##  sample_id  marker_id sequence  count status sample info  ident ident_z
##  <chr>    <chr>   <chr>    <int> <chr>  <chr>  <chr> <dbl> <dbl>
## 1 S01     CSP     CCCAAATG…  2481  pass  3D7_D… 1_1  1    1.05
## 2 S01     CSP     CCCCAATG…  2397  pass  3D7_D… 1_1  0.892 -0.843
## 3 S01     TRAP    TGGGTGAA…  2444  pass  3D7_D… 1_1  1     0.949
## 4 S01     TRAP    TGGGTGAA…  2432  pass  3D7_D… 1_1  0.958 -0.949
## 5 S02     CSP     CCCAAATG…   496  pass  3D7_D… 1_10  1     1.05
## 6 S02     CSP     CCCCAATG…  4378  pass   3D7_D… 1_10  0.892 -0.843
## 7 S02     TRAP    TGGGTGAA…   495  pass  3D7_D… 1_10  1    0.949
## 8 S02     TRAP    TGGGTGAA…  4374  pass  3D7_D… 1_10  0.958 -0.949
## 9 S03     CSP     CCCAAATG…    40  pass  3D7_D… 1_100  1    1.05
## 10 S03     CSP     CCCCAATG…  4778  pass  3D7_D… 1_100  0.892 -0.843
## 11 S03     TRAP    TGGGTGAA…   63  pass  3D7_D… 1_100  1    0.949
## 12 S03     TRAP    TGGGTGAA…  4805  pass  3D7_D… 1_100  0.958 -0.949
## 13 S04     CSP     CCCAAATG…    9 pass  3D7_D… 1_500  1    1.05
## 14 S04     CSP     CCCCAATG…  4926 pass  3D7_D… 1_500  0.892 -0.843
## 15 S04     TRAP    TGGGTGAA…    8 pass  3D7_D… 1_500  1    0.949
## 16 S04     TRAP    TGGGTGAA…  4925  pass  3D7_D… 1_500  0.958 -0.949
## 17 S05     CSP     CCCCAATG…  4737  pass  3D7_D… 1_10…  0.892 -0.843
## 18 S05     TRAP    TGGGTGAA…    5 pass  3D7_D… 1_10…  1    0.949
## 19 S05     TRAP    TGGGTGAA…  4948 pass  3D7_D… 1_10…  0.958 -0.949



The main outputs for the annotate_seq_tbl function are:

• the seq_ann_tbl.rds table: the annotated amplicon haplotype sequence table in RDS format includes sample_id, marker_id, sequence (haplotype sequence), count (read counts), status (inferred haplotype status), sample, info, ident (sequence similarity), ident_z (standardized sequence similarity).

The amplicon sequence haplotype status includes the following states:

• **pass:** the amplicon sequences haplotype has passed all quality control checks,
• **
low_sample_count, failed:** the sample read count lower than the minimum number of read per sample per marker,
• **
low_asv_count, failed:** the amplicon sequence haplotype counts lower than the minimum number of read for each haplotype,
• **
low_asv_freq, failed:** the amplicon sequence haplotype frequency lower than the minimum haplotype frequency,
• **low_ident, failed:** the amplicon sequence haplotype similarity (after being mapped to the reference) is lower than the minimum sequence similarity,
• **
low_ident_z, failed:** the standardized amplicon sequence haplotype similarity (after being mapped to the reference) lower than the minimum standardized sequence similarity,
• **chimera, failed:** the sequence is a chimera of reads,
• **multiple, failed:** multiple fail types.

The sequence_filter function is mainly optimizing amplicon haplotype calling and uses several parameters, such as sample missingness, amplicon marker missingness, the number of alleles at a locus, as well as variant heterozygosity and MAF in a given dataset. This function is implemented with Biostrings, magrittr, withr, GenomicRanges, VariantAnnotation, S4Vectors, IRanges, and tidyverse R packages.

*# Haplotype filtering*
seq_flt_tbl <- sequence_filter(seq_ann_tbl = seq_ann_tbl,
                   sample_manifest = sample_manifest,
                   marker_info = marker_info,
                   output_dir = run_dir,
                   vcf_output_dir = file.path(run_dir, 'vcf'),
                   sample_med_He = 0.1
                  )
seq_flt_tbl

## # A tibble: 19 × 11
##  sample_id marker_id sequence count masked status ident haplo…^1^ frequ…^2^ sample
##  <chr>   <chr>   <chr>   <int> <lgl>  <chr> <dbl> <chr>  <dbl>  <chr>
## 1 S01    CSP    CCCAAAT…  2481 FALSE  pass  1    CSP-2  0.509  3D7_D…
## 2 S01    CSP    CCCCAAT…  2397 FALSE  pass  0.892 CSP-1   0.491  3D7_D…
## 3 S01    TRAP    TGGGTGA…  2444 FALSE  pass  1    TRAP-1  0.501  3D7_D…
## 4 S01    TRAP    TGGGTGA…  2432 FALSE  pass   0.958 TRAP-2  0.499  3D7_D…
## 5 S02    CSP     CCCAAAT…  496 FALSE  pass  1    CSP-2  0.102  3D7_D…
## 6 S02    CSP     CCCCAAT…  4378 FALSE  pass  0.892 CSP-1   0.898  3D7_D…
## 7 S02    TRAP    TGGGTGA…  495 FALSE  pass  1    TRAP-1  0.102  3D7_D…
## 8 S02    TRAP    TGGGTGA…  4374 FALSE  pass  0.958 TRAP-2  0.898  3D7_D…
## 9 S03    CSP    CCCAAAT…   40 FALSE  pass  1   CSP-2  0.0083 3D7_D…
## 10 S03    CSP     CCCCAAT… 4778 FALSE  pass  0.892 CSP-1  0.992  3D7_D…
## 11 S03    TRAP   TGGGTGA…   63 FALSE  pass  1   TRAP-1 0.0129 3D7_D…
## 12 S03    TRAP   TGGGTGA… 4805 FALSE  pass  0.958 TRAP-2 0.987  3D7_D…
## 13 S04     CSP    CCCAAAT…   9 FALSE  pass  1   CSP-2  0.00182 3D7_D…
## 14 S04    CSP    CCCCAAT… 4926 FALSE  pass  0.892 CSP-1  0.998  3D7_D…
## 15 S04    TRAP    TGGGTGA…   8 FALSE  pass  1   TRAP-1  0.00162 3D7_D…
## 16 S04      TRAP   TGGGTGA… 4925 FALSE  pass  0.958 TRAP-2  0.998  3D7_D…
## 17 S05    CSP    CCCCAAT… 4737 FALSE  pass  0.892 CSP-1  1     3D7_D…
## 18 S05     TRAP   TGGGTGA…   5 FALSE  pass   1   TRAP-1 0.00101 3D7_D…
## 19 S05    TRAP   TGGGTGA…  4948 FALSE  pass  0.958 TRAP-2 0.999  3D7_D…
## #… with 1 more variable: info <chr>, and abbreviated variable names
## #   ^1^​haplotype, ^2^​frequency



The main outputs for the sequence_filter function are:

• the seq_flt_tbl.rds table: the final amplicon haplotype sequence table in RDS format which includes sample_id, marker_id, sequence (haplotype sequence), count (read counts), masked (TRUE indicates the sequence contains variants that might be errors, and replaces the nucleotide with the reference genome nucleotide), status (haplotype status, all pass), ident (sequence similarity), haplotype (a named panel of haplotypes for each marker), frequency (within-sample haplotype frequency for each marker), sample, info.

### Data visualization

The generate_report function generates a HTML report including various summary data visualizations. The function is based on Biostrings, Rmarkdown, plyr, DT, plotly, viridisLite, stats, flextable, ComplexHeatmap, scales, heatmaply, digest, tidyselect, data.table, DECIPHER, htmltools, pheatmap, ape, randomcoloR, ggtree, ggstance, and tidyverse R packages. The HTML summary report for the synthetic *P. falciparum* dataset is available from Figshare ( https://doi.org/10.6084/m9.figshare.21739121). ^27^

*# Data visualization*
generate_report(sample_manifest = sample_manifest,
          marker_info = marker_info,
          demultiplexed = demultiplexed,
          flt_reads = flt_reads,
          sub_reads = sub_reads,
          seq_ann_tbl = seq_ann_tbl,
          seq_flt_tbl = seq_flt_tbl,
          report_output_dir = file.path(run_dir, 'report'))

## ---- generating report ----

## report save to "runs/report/Report.html"



We also include the process_run function that calls all the functions, making it easy to get all results.

*# Create the analysis directory*
process_run_dir <- "runs_process"
dir.create("./runs_process")

*# Input data*
reads_1 <- example_data$reads_1
reads_2 <- example_data$reads_2
sample_manifest <- example_data$sample_manifest
marker_info <- example_data$marker_info

*# Fast run*
process_run(reads_1 = reads_1,
       reads_2 = reads_2,
       sample_manifest = sample_manifest,
       marker_info = marker_info,
       run_dir = process_run_dir,
       min_marker_count = 500,       sample_med_He = 0.1)

## ---- processing run ----

## ---- demultiplexing ----

## ---- filter and trim ----

## ---- downsampling ----

## ---- ASV estimation ----

## ---- filtering haplotype sequence ----

## ---- generating report ----



### SARS-CoV-2 AmpSeq data

**Figure 3.**
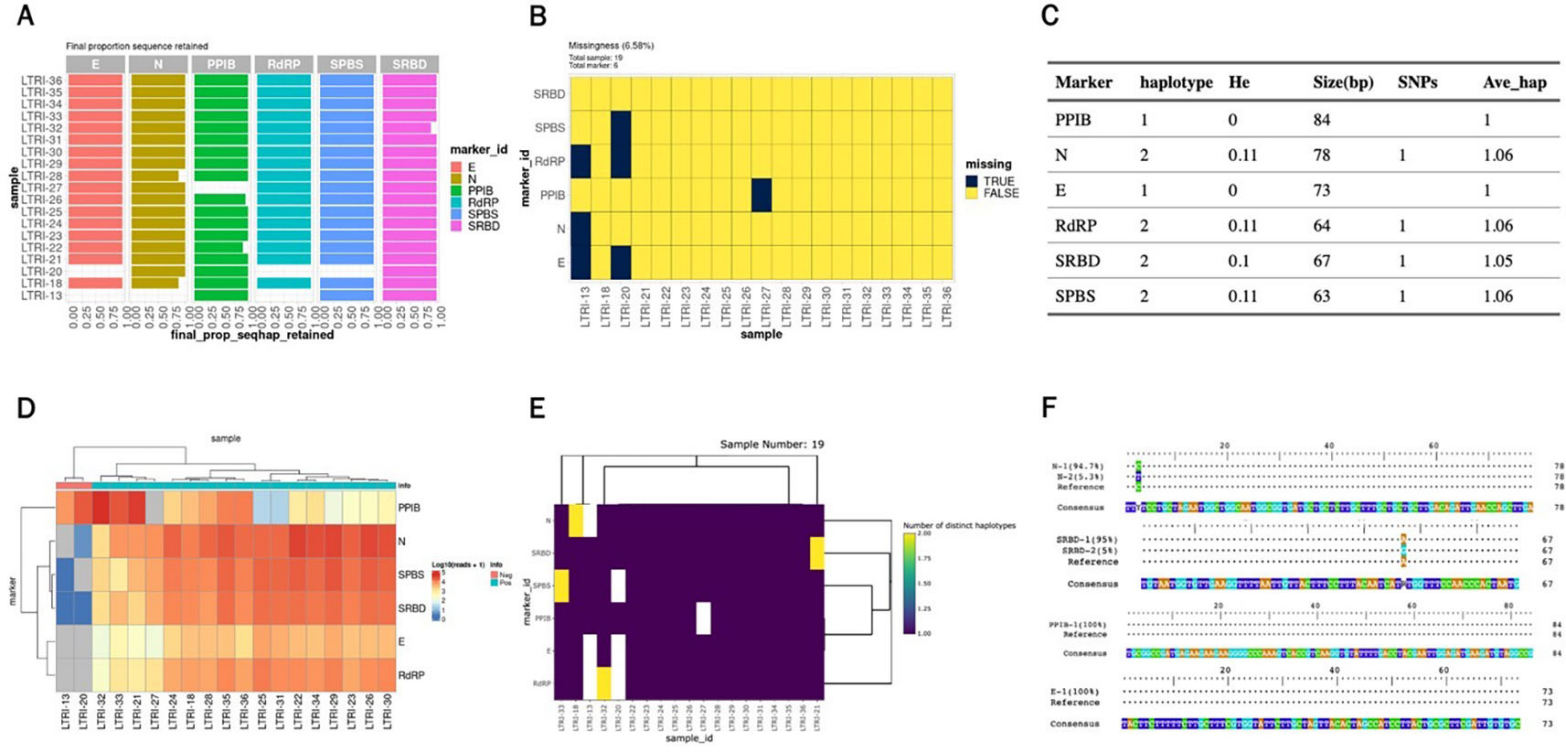
Example visualisations represented in output from the AmpSeqR HTML report. A. The proportion of sequences that are finally retained. B. Missing values in the dataset. C. Haplotype diversity metrics for amplicon markers in the dataset. D. Read counts for amplicon markers and all samples in the dataset. E. Number of unique haplotypes detected per amplicon per sample. F. Visualisation of haplotype and reference sequences.

For the SARS-CoV-2 AmpSeq data, the paired-end FASTQ data is already demultiplexed by sample. We first demultiplexed by marker and then run AmpSeqR from the filter and trim step as follows. Some output from the HTML report for the SARS-CoV-2 dataset is shown in Figure 3.

*# Input data*
sample_manifest <- read_csv("Covid19/sample_manifest.csv", col_types = cols())
marker_info <- read_csv("Covid19/marker_info.csv", col_types = cols(start = col_integer()))

*# The paired-end FASTQ data already demultiplexed*
demultiplexed <- readRDS("Covid19/demultiplex.rds")

*# Create the analysis directory*
dir.create("./runs")

*# Filter and trim reads*
flt_reads <- demultiplexed %>%
  dada_filter(output_dir = run_dir,
         output_sub_dir = file.path(run_dir, 'filter'))

*# Downsampling reads*
sub_reads <- flt_reads %>%
  downsample_reads(output_dir = run_dir,
            output_sub_dir = file.path(run_dir, 'downsample'),
            min_read_count = 0,
            count_col = 'n_out')

*# Construct amplicon sequence variant table (ASV) table*
seq_tbl <- sub_reads %>%
  dada_seq_tbl(output_dir = run_dir)

*# Haplotype inference*
seq_ann_tbl <- seq_tbl %>%
  annotate_seq_tbl(marker_info = marker_info,
            output_dir = run_dir,
            min_marker_count = 0,
            min_asv_count = 0)

*# Haplotype filtering*
seq_flt_tbl <- sequence_filter(seq_ann_tbl = seq_ann_tbl,
                   sample_manifest = sample_manifest,
                   marker_info = marker_info,
                   output_dir = run_dir,
                   vcf_output_dir = file.path(run_dir, 'vcf'),
                   max_sm_miss = 1,
                   max_marker_miss = 1,
                   min_homo_rep = NULL,
                   terminal_region_len = NULL
                  )

*# Data visualization*
generate_report(sample_manifest = sample_manifest,
          marker_info = marker_info,
          demultiplexed = demultiplexed,
          flt_reads = flt_reads,
          sub_reads = sub_reads,
          seq_ann_tbl = seq_ann_tbl,
          seq_flt_tbl = seq_flt_tbl,
          report_output_dir = file.path(run_dir, 'report'))



### *Plasmodium falciparum* AmpSeq data

**Figure 4.**
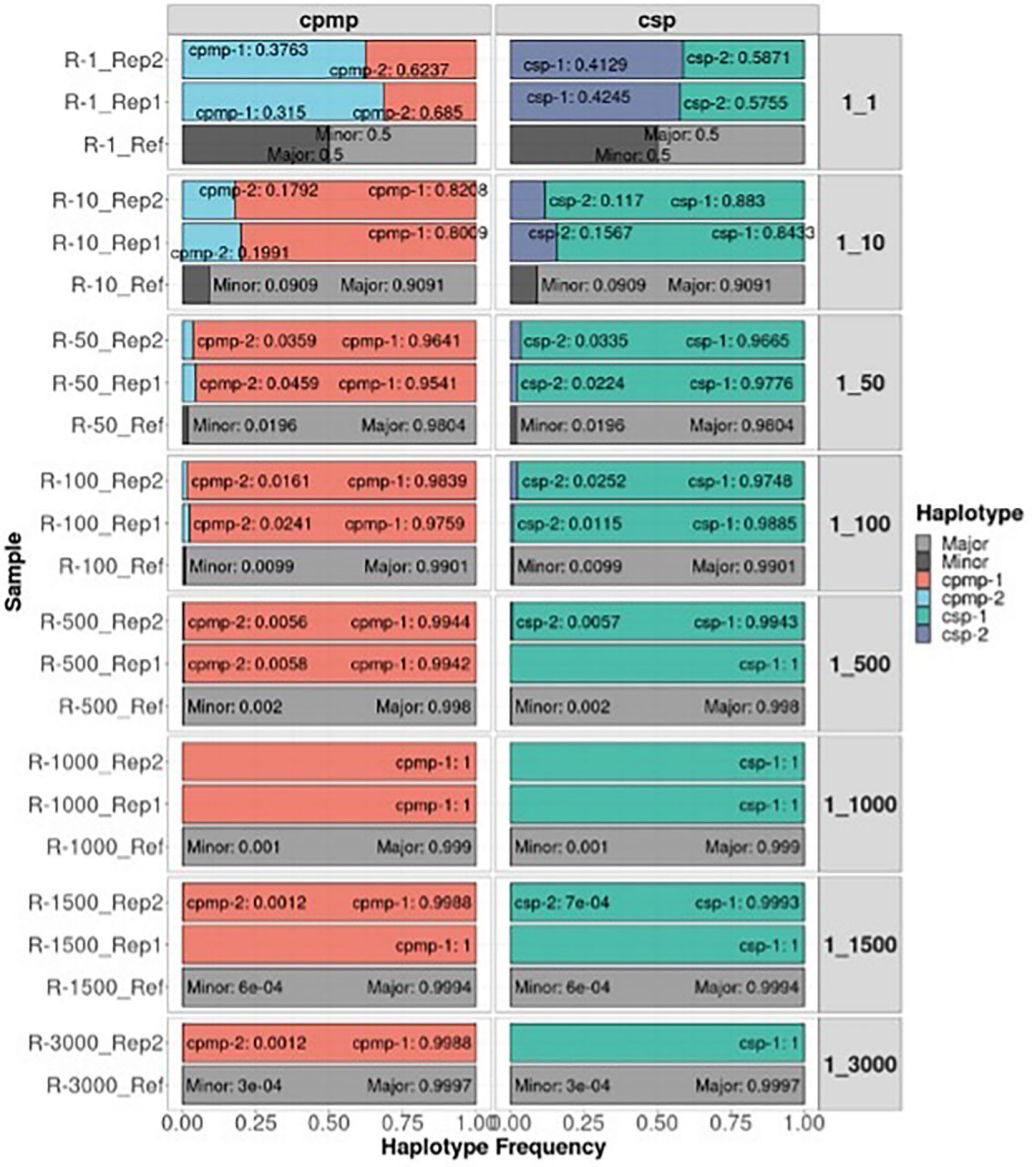
The detectability of the minor clone in *P. falciparum* AmpSeq data using *CPMP* and *CSP* markers across mixture ratios (1:1 to 1:3000). The x-axis represents the haplotype frequency, and the y-axis represents the sample. Colored by haplotype and labelled by haplotype and haplotype frequency. The grey bar represents a reference for different defined mixture proportions.

The detectability of the minor clone of the *CPMP* and *CSP* amplicon markers under different mixture ratios of *P. falciparum* is shown in Figure 4. The correct minor haplotype was detected in mixture proportions from 1:1 to 1:3000 in most mixtures and detected haplotype frequencies similar to the defined mixture proportion. These results demonstrate that AmpSeqR can detect low-frequency haplotypes accurately.

## Conclusions

We present the AmpSeqR R package for analysis of AmpSeq data which was primarily developed for the analysis of infectious diseases. The pipeline offers various useful steps including data pre-processing, amplicon sequence variants (ASVs) estimation, data post-processing, data visualization and includes several parameters to filter noise reads and improve the accuracy of the detected haplotype. AmpSeqR allows users to easily analyze the AmpSeq data and generate an HTML report including various summary data visualizations. Additionally, the one-line commands for processing the data do not require extensive R knowledge which provides a user-friendly experience for R users of all levels of experience.

## Data Availability

Figshare: Underlying data for ‘AmpSeqR: an R package for amplicon deep sequencing data analysis’. https://doi.org/10.6084/m9.figshare.21739121.v2. ^27^ Data are available under the terms of the Creative Commons Attribution 4.0 International license (CC-BY 4.0). NCBI GEO: A multiplexed, next-generation sequencing platform for high-throughput detection of SARS-CoV-2. Accession number GSE160031; https://identifiers.org/geo:GSE160031 NCBI Genome: SARS-CoV-2 isolate Wuhan-Hu-1, complete genome. Accession number NC_045512; https://www.ncbi.nlm.nih.gov/nuccore/NC_045512.2 BioProject: Amplicon deep sequencing of multi-clonal *Plasmodium falciparum* infections. Accession number PRJNA381546. https://identifiers.org/bioproject:PRJNA381546

## Software availability

Source code available from: https://github.com/bahlolab/AmpSeqR

Archived source code at time of publication: https://doi.org/10.5281/zenodo.7580184. ^16^

License: GNU General Public License v3.0
