# Supplementary material for: AmpSeqR: an R package for amplicon deep sequencing data analysis
Source: F1000Res. 2025 Oct 17;12:327. Originally published 2023 Mar 23. [Version 2] doi: 10.12688/f1000research.129581.2 (PMC11584457; doi:10.12688/f1000research.129581.2)
Supplement: Peer review history

# Reviewer report: Reviewer response for version 2

**DOI:** 10.5256/f1000research.189562.r425010

**Published:** 2025-11-18

**Authors:** Eduardo Castro-Nallar

**Affiliations:** 1 Universidad de Talca, Talca, Maule Region, Chile

Thank you for adding a new table and for updating existing table 2.

Are the conclusions about the tool and its performance adequately supported by the findings presented in the article?

Partly

Is the rationale for developing the new software tool clearly explained?

Partly

Is the description of the software tool technically sound?

Yes

Are sufficient details of the code, methods and analysis (if applicable) provided to allow replication of the software development and its use by others?

Yes

Is sufficient information provided to allow interpretation of the expected output datasets and any results generated using the tool?

Yes

Reviewer Expertise:

My area of research is microbial genomics. I work primarily with environmental microbial communities understanding their ecology and evolution through a genetics/genomic lens. I have occasionally worked on software development that focuses on microbial genomics. I have also benchmarked tools developed for metagenomic and amplicon sequencing analysis.

I confirm that I have read this submission and believe that I have an appropriate level of expertise to confirm that it is of an acceptable scientific standard.

# Reviewer report: Reviewer response for version 2

**DOI:** 10.5256/f1000research.189562.r425009

**Published:** 2025-11-11

**Authors:** Cristian Koepfli, Gustavo Da Silva

**Affiliations:** 1 University of Notre Dame, Notre Dame, IN, USA; 2 Biological Sciences, University of Notre Dame Eck Institute for Global Health (Ringgold ID: 550321), Notre Dame, Indiana, USA

We tested the workflow again and now it works perfectly. We tested some of our own samples; this is indeed a very useful pipeline.

 The authors also addressed my other, minor comments.

Are the conclusions about the tool and its performance adequately supported by the findings presented in the article?

Partly

Is the rationale for developing the new software tool clearly explained?

Partly

Is the description of the software tool technically sound?

Yes

Are sufficient details of the code, methods and analysis (if applicable) provided to allow replication of the software development and its use by others?

No

Is sufficient information provided to allow interpretation of the expected output datasets and any results generated using the tool?

Yes

Reviewer Expertise:

Malaria epidemiology, diagnosis, and genomics

We confirm that we have read this submission and believe that we have an appropriate level of expertise to confirm that it is of an acceptable scientific standard.

# Reviewer report: Reviewer response for version 1

**DOI:** 10.5256/f1000research.142271.r333941

**Published:** 2024-11-22

**Authors:** Patrick Murigu Kamau Njage

**Affiliations:** 1 Technical University of Denmark, Lyngby, Denmark

Though this is an amazing package that may solve analysis and reporting bottlenecks in amplicon sequencing data analysis, the newly developed functions that are time intensive may need to be benchmarked against existing tools in terms of computation time if this is possible. 

 R packages are listed without a clear in the section "Operation" and "Data visualization" without a clear link to their roles. An appendix that lists the role of each of the R packages rather than just listing them in the text would make the package more reproducible.

 The two sentences " *PCA is also performed to visualize*

*the sample structure. The process is handled using the Biostrings, Rmarkdown, plyr, DT, plotly, viridisLite, stats,*

*flextable, ComplexHeatmap,* *22* *scales, heatmaply, digest, tidyselect, data.table, DECIPHER, htmltools, pheatmap*

*(RRID:SCR_016418), ape, randomcoloR, ggtree,* *23* *ggstance, and several tidyverse R packages.*" may deceptively imply that all the packages listed in the second sentence are for handling PCAs. 

 Figure 4 caption needs to include a minimum amount of information for the reader to understand it without a deep search from the text. What are the mixture ratios?

Are the conclusions about the tool and its performance adequately supported by the findings presented in the article?

Yes

Is the rationale for developing the new software tool clearly explained?

Partly

Is the description of the software tool technically sound?

Yes

Are sufficient details of the code, methods and analysis (if applicable) provided to allow replication of the software development and its use by others?

Partly

Is sufficient information provided to allow interpretation of the expected output datasets and any results generated using the tool?

Yes

Reviewer Expertise:

Infectius disease microbial genomics and data analysis.

I confirm that I have read this submission and believe that I have an appropriate level of expertise to confirm that it is of an acceptable scientific standard.

# Response

**Published:** 2025-10-09

**Authors:** Melanie Bahlo

**Affiliations:** The Walter & Eliza Hall Institute of Medical Research, Australia

**Comment #1: **Though this is an amazing package that may solve analysis and reporting bottlenecks in amplicon sequencing data analysis, the newly developed functions that are time intensive may need to be benchmarked against existing tools in terms of computation time if this is possible.

**Response: **We thank the reviewer for this suggestion. Although direct benchmarking with other AmpSeq tools is not straightforward due to differences in scope and outputs, we have added a new table (Table 3) summarizing the runtimes of all major AmpSeqR functions across the three example datasets used in our Use Cases section, as well as one large in-house dataset. This provides an overview of performance and highlights how runtime scales with dataset size.

 Table 3. Runtime performance of AmpSeqR functions across datasets of different size and complexity. All values are reported in seconds.

**Comment #2: **R packages are listed without a clear in the section "Operation" and "Data visualization" without a clear link to their roles. An appendix that lists the role of each of the R packages rather than just listing them in the text would make the package more reproducible.

**Response: **We thank the reviewer for this valuable suggestion. We have updated Table 2 (Description of the AmpSeqR major functions) to include an additional column, “Key R packages implemented.” This column lists the main packages used for each function, providing a clear link between the software dependencies and their roles in the workflow.

**Comment #3: **The two sentences "PCA is also performed to visualize the sample structure. The process is handled using the Biostrings, Rmarkdown, plyr, DT, plotly, viridisLite, stats, flextable, ComplexHeatmap,22 scales, heatmaply, digest, tidyselect, data.table, DECIPHER, htmltools, pheatmap (RRID:SCR_016418), ape, randomcoloR, ggtree,23 ggstance, and several tidyverse R packages." may deceptively imply that all the packages listed in the second sentence are for handling PCAs.

**Response: **We thank the reviewer for this comment. We have revised the text for clarity, as follows:

 “More broadly, the data visualization and reporting steps in AmpSeqR are implemented using a range of R packages, including Biostrings, Rmarkdown … and several tidyverse R packages.

**Comment #4: **Figure 4 caption needs to include a minimum amount of information for the reader to understand it without a deep search from the text. What are the mixture ratios?

**Response: **We thank the reviewer for pointing this out. We have revised the caption of Figure 4 to provide sufficient context. The revised caption is provided below:

 “Figure 4. The detectability of the minor clone in *P. falciparum* AmpSeq data using *CPMP* and *CSP* markers across mixture ratios (1:1 to 1:3000). The x-axis represents the haplotype frequency, and the y-axis represents the sample. Colored by haplotype and labelled by haplotype and haplotype frequency. The grey bar represents a reference for different defined mixture proportions.”

# Reviewer report: Reviewer response for version 1

**DOI:** 10.5256/f1000research.142271.r330664

**Published:** 2024-11-12

**Authors:** Cristian Koepfli, Gustavo Da Silva

**Affiliations:** 1 University of Notre Dame, Notre Dame, IN, USA; 2 Biological Sciences, University of Notre Dame Eck Institute for Global Health (Ringgold ID: 550321), Notre Dame, Indiana, USA

Jiru Han and colleagues describe a new bioinformatic pipeline for the analysis of amplicon sequencing data. The pipeline incorporates several existing bioinformatic tools and adds new ones, including a comprehensive visual report. One of the aims is to have a pipeline that is easier to use for non-bioinformaticians compared to other pipelines. 

 As a disclaimer to my review, I was closely involved in the development of HaplotypeR, and the generation of dataset 3 that was used in the current manuscript to test AmpSeqR. The code was tested by Gustavo da Silva, a student under my supervision.

 Main Comments:

 We were not able to run the code given in the manuscript in the conditions below.

1. The regular run option in AmpSeqR works as expected with its example files. However, when we applied it to our own aplicon sequencing samples for *Plasmodium falciparum *and *Plasmodium vivax*, it encountered an error during the demultiplexing step, where the program sorts the data. We formatted the files to match the example format exactly, but they still failed. We then tried different configurations, such as playing with the data frames and tables, but none of these attempts resolved the issue. Without clear guidance on how to structure non-example data, AmpSeqR's utility for processing diverse datasets is significantly limited.
2. Additionally, the fast run option did not produce any output files for us. It fails even with the example files, and it also doesn’t work with any of our custom samples - expected after the problems observed above. It would be helpful if the fast run generated output files at each step, rather than only at the final report stage, as this would make it easier to identify where problems might be occurring. Such files would be helpful for the regular run as well.
3. Finally, when generating reports, AmpSeqR encounters an error in the ‘train()’ function due to an unused argument, which may be linked to its interactions with ggplot2 or plotly. This error prevents the report from being created, leaving only the ‘seq_flt_tbl’ table in the environment. It happens both with the regular run and the fast run.

The paper states that existing methods have “difficulty in distinguishing between true sequence and PCR sequencing errors and artifacts”. First, I am not sure what is meant by artifacts. The main challenge for any analysis of such types of sequencing typically is the distinction between PCR/sequencing errors and true minority clones present at low frequency within in sample. This is an inherent challenge to any bioinformatic analysis, it is not clear how AmpSeqR overcomes this issue. More discussion on what AmpSeqR does differently compared to other pipelines, and how this is achieved, would be useful. Either the introduction could be expanded, or a proper discussion section could be added.

 Minor comments:

 The writing is sometimes a bit confusing and could be clarified. E.g.:

  

1. Please clarify the following sentences: “and also the ability to multiplex hundreds of samples in a single run, thereby reducing the sequencing costs. AmpSeq is highly scalable and cheap, permitting low-cost data generation for large sample sizes. It can also be multiplexed to further increase resolution.”

I believe the first ‘multiplexed’ refers to multiplexing of samples, while the second refers to multiplexing of markers.

1. I am a bit surprised about SARS-CoV-2 being highlighted in the introduction of an amplicon sequencing paper. A lot (though not all) of sequencing studies sequenced full genomes, which is different from amplicons of a few hundred bp. Likely, AmpSeqR is also useful for viral WGS data, but it would be good to discuss this more specifically.
2. Just following the section on SARS-CoV-2, a sentence in the introduction states that “AmpSeq methods are also widely used in infectious disease characterization and surveillance studies”. Given covid-19 is an infectious disease, this sentence is confusing. Maybe change to “AmpSeq methods are widely used in characterization and surveillance studies of many other infectious diseases”.
3. The meaning of the term relapse as used in P. vivax studies might not be known to readers. The term is used for different processes in different diseases (compare e.g. to cancer relapse).

Typo:

 “well as assigning the sequence to each” > assigns

Are the conclusions about the tool and its performance adequately supported by the findings presented in the article?

Partly

Is the rationale for developing the new software tool clearly explained?

Partly

Is the description of the software tool technically sound?

Yes

Are sufficient details of the code, methods and analysis (if applicable) provided to allow replication of the software development and its use by others?

No

Is sufficient information provided to allow interpretation of the expected output datasets and any results generated using the tool?

Yes

Reviewer Expertise:

Malaria epidemiology, diagnosis, and genomics

We confirm that we have read this submission and believe that we have an appropriate level of expertise to confirm that it is of an acceptable scientific standard, however we have significant reservations, as outlined above.

# Response

**Published:** 2025-10-09

**Authors:** Melanie Bahlo

**Affiliations:** The Walter & Eliza Hall Institute of Medical Research, Australia

**Main Comments:**

 We were not able to run the code given in the manuscript in the conditions below.

**Comment #1: **The regular run option in AmpSeqR works as expected with its example files. However, when we applied it to our own aplicon sequencing samples for Plasmodium falciparum and Plasmodium vivax, it encountered an error during the demultiplexing step, where the program sorts the data. We formatted the files to match the example format exactly, but they still failed. We then tried different configurations, such as playing with the data frames and tables, but none of these attempts resolved the issue. Without clear guidance on how to structure non-example data, AmpSeqR's utility for processing diverse datasets is significantly limited.

**Response: **We thank the reviewer for this comment. Example input data are shown in the Use Cases (Installing the package) section to illustrate the correct file structure. In addition, we have revised this section to provide more detail on the expected structure of the sample barcodes file and the target amplicon detail file, including required columns, data types, and formatting, as follows:

 “The sample barcodes file should include sample_id, barcode_fwd, barcode_rev, sample (sample name, can be the same as sample_id), info (e.g., sample type). All columns should be in character format, and barcode_fwd and barcode_rev must be consistent DNA-encoded barcodes of equal length. The target amplicon detail file should include marker_id, primer_fwd, primer_rev, seq (reference sequence), chrom (chromosome), start (reference sequence start position), end (reference sequence end position). All columns should be in character format, except start and end, which must be numeric. This file should be set up in a text editor or excel and saved as text CSV format (*.csv) file.”

**Comment #2: **Additionally, the fast run option did not produce any output files for us. It fails even with the example files, and it also doesn’t work with any of our custom samples - expected after the problems observed above. It would be helpful if the fast run generated output files at each step, rather than only at the final report stage, as this would make it easier to identify where problems might be occurring. Such files would be helpful for the regular run as well.

**Response: **We thank the reviewer for highlighting this issue. The process_run fast run function is designed to generate output files at each step, including demultiplexing, filtering and trimming, downsampling, ASV estimation, haplotype sequence filtering, and report generation. We identified that the failure stemmed partly from dependency updates in some R packages since the initial release. AmpSeqR has now been updated for compatibility with R versions 4.4.1 and 4.5, and we have re-tested it on multiple computers using the three example datasets described in the Use Cases section as well as additional large in-house datasets. In all cases, both the regular run and fast run options now work successfully.

 To improve robustness and reproducibility, AmpSeqR will be actively maintained going forward. Users are encouraged to report any issues or bugs via the contact email provided in the package documentation and on the GitHub repository.

**Comment #3: **Finally, when generating reports, AmpSeqR encounters an error in the ‘train()’ function due to an unused argument, which may be linked to its interactions with ggplot2 or plotly. This error prevents the report from being created, leaving only the ‘seq_flt_tbl’ table in the environment. It happens both with the regular run and the fast run.

**Response: **We thank the reviewer for this comment. We identified that the issue stemmed in part from dependency updates in some R packages since the initial release. We have corrected this by updating the reporting functions to ensure compatibility with the latest package versions. AmpSeqR has also been updated to be compatible with the latest R versions (4.4.1 and 4.5) and tested on the three example datasets described in the Use Cases section as well as additional large in-house datasets. In all cases, both the regular run and fast run options now work successfully.

**Comment #4: **The paper states that existing methods have “difficulty in distinguishing between true sequence and PCR sequencing errors and artifacts”. First, I am not sure what is meant by artifacts. The main challenge for any analysis of such types of sequencing typically is the distinction between PCR/sequencing errors and true minority clones present at low frequency within in sample. This is an inherent challenge to any bioinformatic analysis, it is not clear how AmpSeqR overcomes this issue. More discussion on what AmpSeqR does differently compared to other pipelines, and how this is achieved, would be useful. Either the introduction could be expanded, or a proper discussion section could be added.

**Response: **We thank the reviewer for this comment. By “artifacts,” we refer to false haplotypes arising from PCR or sequencing errors, such as chimeric reads or spurious low-frequency variants. We agree that distinguishing true minority clones, especially very low-frequency variants, from sequencing errors is a significant challenge. AmpSeqR improve this by combining DADA2’s error-correction framework with several additional haplotype filtering steps, including removal of chimeras, correction of indels and homopolymers, checks of sequence similarity to the reference, evaluation of variant heterozygosity, and dataset-level checks. These approaches are described in the Data Post-processing section, and we have now revised the Introduction to clarify this point as follows:

 “The pipeline integrates several R packages as well as newly developed functions to filter out sequencing noise, including the removal of chimeric reads, correction of indels and homopolymers, checks of sequence similarity against the reference, evaluation of variant heterozygosity, and dataset-level checks, all of which improve the accuracy of sequence data detection.”

**Minor comments:**

 The writing is sometimes a bit confusing and could be clarified. E.g.:

**Comment #1: **Please clarify the following sentences: “and also the ability to multiplex hundreds of samples in a single run, thereby reducing the sequencing costs. AmpSeq is highly scalable and cheap, permitting low-cost data generation for large sample sizes. It can also be multiplexed to further increase resolution.” I believe the first ‘multiplexed’ refers to multiplexing of samples, while the second refers to multiplexing of markers.

**Response: **Thanks for the suggestion. We have revised it as follows: “Amplicon sequencing (AmpSeq) is increasingly used in biological and medical studies, offering high coverage for the detection of rare variants and multiplexing capacity that reduces sequencing costs. It is highly scalable and cost-effective, enabling data generation for large sample sizes, and can be further multiplexed at the marker level to increase resolution.”

**Comment #2: **I am a bit surprised about SARS-CoV-2 being highlighted in the introduction of an amplicon sequencing paper. A lot (though not all) of sequencing studies sequenced full genomes, which is different from amplicons of a few hundred bp. Likely, AmpSeqR is also useful for viral WGS data, but it would be good to discuss this more specifically.

**Response: **We thank the reviewer for this comment. Our intention in highlighting SARS-CoV-2 was to provide a recent example where amplicon sequencing was widely applied. To avoid confusion, we have revised the Introduction to clarify this distinction. 

 “Severe acute respiratory syndrome coronavirus 2 (SARS-CoV-2), the causative agent of coronavirus disease 2019 (COVID-19), continues to pose a serious threat to the global population health. While whole-genome sequencing was commonly used for SARS-CoV-2, targeted AmpSeq of defined genome regions was also widely applied, particularly in diagnostic and surveillance settings, to monitor viral presence and detect emerging variants.”

**Comment #3: **Just following the section on SARS-CoV-2, a sentence in the introduction states that “AmpSeq methods are also widely used in infectious disease characterization and surveillance studies”. Given covid-19 is an infectious disease, this sentence is confusing. Maybe change to “AmpSeq methods are widely used in characterization and surveillance studies of many other infectious diseases”.

**Response:** We thank the reviewer for this comment. We revised these as suggested.

**Comment #4: **The meaning of the term relapse as used in P. vivax studies might not be known to readers. The term is used for different processes in different diseases (compare e.g. to cancer relapse).

**Response:** We thank the reviewer for this comment. We have now revised to clarify this point as follows:

 “AmpSeq methods are widely used in characterization and surveillance studies of many other infectious diseases (e.g., malaria infection) to monitor the emergence and spread of drug resistance, to examine population structure, to study relapse in malaria caused by activation of dormant liver-stage hypnozoites, to estimate clearance rates and to track within-host dynamics in longitudinal studies.”

**Comment #5: **Typo:

 “well as assigning the sequence to each” > assigns

**Response: **Thanks for pointing out this typo. We revised this as suggested.

# Reviewer report: Reviewer response for version 1

**DOI:** 10.5256/f1000research.142271.r319368

**Published:** 2024-11-09

**Authors:** Eduardo Castro-Nallar

**Affiliations:** 1 Universidad de Talca, Talca, Maule Region, Chile

The rationale for developing the new software tool is partly explained, as the value of amplicon sequencing and its importance in diverse scientific disciplines is clear. However, the rationale for developing this tool in the light of many other published tools with similar functionality is not clear.

 Regarding the performance. The manuscript does not mention compute time per sample or dataset so that it's difficult to assess whether someone should select this tool over another. Likewise, there's no comparative benchmark study with other published tools.

 In summary, this is a wrapper tool that at its core uses DADA2 to infer amplicon sequence variants and offers some handy functions to conduct steps before and after DADA2's inference.

Are the conclusions about the tool and its performance adequately supported by the findings presented in the article?

Partly

Is the rationale for developing the new software tool clearly explained?

Partly

Is the description of the software tool technically sound?

Yes

Are sufficient details of the code, methods and analysis (if applicable) provided to allow replication of the software development and its use by others?

Yes

Is sufficient information provided to allow interpretation of the expected output datasets and any results generated using the tool?

Yes

Reviewer Expertise:

My area of research is microbial genomics. I work primarily with environmental microbial communities understanding their ecology and evolution through a genetics/genomic lens. I have occasionally worked on software development that focuses on microbial genomics. I have also benchmarked tools developed for metagenomic and amplicon sequencing analysis.

I confirm that I have read this submission and believe that I have an appropriate level of expertise to confirm that it is of an acceptable scientific standard, however I have significant reservations, as outlined above.

# Response

**Published:** 2025-10-09

**Authors:** Melanie Bahlo

**Affiliations:** The Walter & Eliza Hall Institute of Medical Research, Australia

**Comment #1:** Regarding the performance. The manuscript does not mention compute time per sample or dataset so that it's difficult to assess whether someone should select this tool over another. Likewise, there's no comparative benchmark study with other published tools. In summary, this is a wrapper tool that at its core uses DADA2 to infer amplicon sequence variants and offers some handy functions to conduct steps before and after DADA2's inference.

**Response: **We thank the reviewer for this helpful comment and for raising this important point. While direct benchmarking against other published AmpSeq tools is challenging due to differences in pipeline scope, input requirements, and outputs, we agree that reporting runtime is essential for assessing performance. To address this, we have added a new table (Table 3) summarizing the runtimes of all major AmpSeqR functions across the three example datasets presented in the Use Cases section, as well as one large in-house dataset. This provides an overview of performance and illustrates how runtime scales with dataset size.

 Table 3. Runtime performance of AmpSeqR functions across datasets of different size and complexity. All values are reported in seconds.

 We also acknowledge that AmpSeqR leverages DADA2 for amplicon sequence variant inference, but it extends beyond being a simple wrapper by integrating demultiplexing, trimming, downsampling, chimera/variant filtering, VCF export, and automated visualization and reporting into a single, user-friendly workflow. 

 To improve clarity, we have updated Table 2 (Description of the AmpSeqR major functions) to include an additional column, “Key R packages implemented.” This explicitly links each function to its supporting packages, providing a clear link between the software dependencies and their roles in the workflow.
